# Naphthalene-2,6-dicarb­oxy­lic acid–1-methyl­pyrrolidin-2-one (1/2)

**DOI:** 10.1107/S1600536810052396

**Published:** 2010-12-24

**Authors:** Bianhua Wu, Ge Peng, Youwei Cheng, Xi Li, Jiyong Liu

**Affiliations:** a Department of Chemical and Biological Engineering, Zhejiang University, Hangzhou 310027, People’s Republic of China; b Chemical Engineering College, Ningbo University of Technology, Ningbo 315016, People’s Republic of China; c Department of Chemistry, Zhejiang University, Hangzhou 310027, People’s Republic of China

## Abstract

The asymmetric unit of the title compound, C_12_H_8_O_4_·2C_5_H_9_NO, contains one half-mol­ecule of naphthalene-2,6-dicarb­oxy­lic acid (NDA) and one mol­ecule of 1-methyl­pyrrolidin-2-one (NMP): the NDA molecules lie on the crystallographic twofold rotation axes. In the crystal, the components are linked by strong O—H⋯O hydrogen bonds and C—H⋯O inter­actions.

## Related literature

For the crystal structure of naphthalene-2,6-dicarb­oxy­lic acid (NDA), see: Kaduk & Golab (1999 ▶). For the crystal structure of *N*-methyl-2-Pyrrolidone (NMP), see: Müller *et al.* (1996 ▶). For the purification of NDA, see: Nagase *et al.* (2004 ▶). For related structures, see: Guo *et al.* (2009 ▶); Dale & Elsegood (2004 ▶).
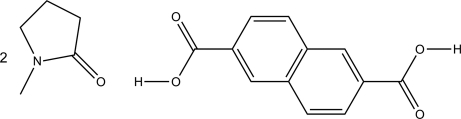


## Experimental

### 

#### Crystal data

C_12_H_8_O_4_·2C_5_H_9_NO
*M* *_r_* = 414.45
Orthorhombic, 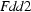

*a* = 19.7306 (11) Å
*b* = 28.7632 (19) Å
*c* = 7.1906 (4) Å
*V* = 4080.8 (4) Å^3^
*Z* = 8
Mo *K*α radiation
μ = 0.10 mm^−1^
*T* = 120 K
0.30 × 0.11 × 0.10 mm

#### Data collection

Oxford Diffraction Xcalibur Atlas Gemini ultra diffractometer
Absorption correction: multi-scan (*CrysAlis PRO*; Oxford Diffraction, 2009 ▶) *T*_min_ = 0.987, *T*_max_ = 0.990
3255 measured reflections
1017 independent reflections
847 reflections with *I* > 2σ(*I*)
*R*_int_ = 0.037

#### Refinement

*R*[*F* ^2^ > 2σ(*F* ^2^)] = 0.037
*wR*(*F* ^2^) = 0.087
*S* = 1.05
1017 reflections
138 parameters
1 restraint
H-atom parameters constrained
Δρ_max_ = 0.17 e Å^−3^
Δρ_min_ = −0.20 e Å^−3^

### 

Data collection: *CrysAlis PRO* (Oxford Diffraction, 2009 ▶); cell refinement: *CrysAlis PRO*; data reduction: *CrysAlis PRO*; program(s) used to solve structure: *SHELXS97* (Sheldrick, 2008 ▶); program(s) used to refine structure: *SHELXL97* (Sheldrick, 12008); molecular graphics: *OLEX2* (Dolomanov *et al.*, 2009 ▶); software used to prepare material for publication: *OLEX2*.

## Supplementary Material

Crystal structure: contains datablocks I, global. DOI: 10.1107/S1600536810052396/su2234sup1.cif

Structure factors: contains datablocks I. DOI: 10.1107/S1600536810052396/su2234Isup2.hkl

Additional supplementary materials:  crystallographic information; 3D view; checkCIF report

## Figures and Tables

**Table 1:** Hydrogen-bond geometry (Å, °)

*D*—H⋯*A*	*D*—H	H⋯*A*	*D*⋯*A*	*D*—H⋯*A*
O1—H1⋯O3	0.82	1.75	2.556 (3)	165
C2—H2⋯O2^i^	0.93	2.48	3.163 (4)	131
C8—H8*A*⋯O2	0.97	2.47	3.311 (4)	145

Symmetry code: (i) 
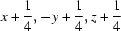
.

**Fig. 1.**
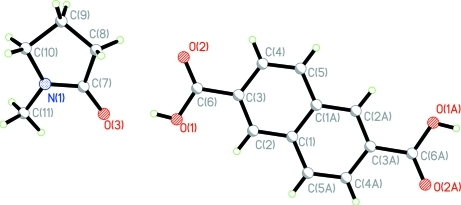
The molecular structure of the Naphthalene-2,6-dicarboxylic acid molecule and one N-Methyl-2-Pyrrolidone molecule of the title compound. Displacement ellipsoids are drawn at the 50% probability level [Symmetry code: A = -x + 0.5, -y + 0.5, z].

**Fig. 2.**
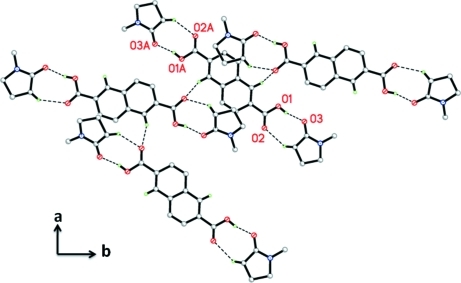
The crystal packing viewed along the c-axis of the title compound, showing the intermolecular O-H···O hydrogen bonds and C-H···O interactions as dashed lines [see Table 1 for details].

## supplementary crystallographic information

### Comment

Naphthalene-2,6-dicarboxylic acid (NDA) is an important monomer for producing polyester and polyurethane materials and liquid crystal polymers (LCP). During the manufacturing process, the impurities in NDA, such as 6-formyl-2-naphthoic acid (FNA), debase the quality of the products dramatically. So the purification of NDA is very important however, this process is difficult (Nagase *et al.*, 2004). Although many methods have been proposed in this field, they are either too complex or not cost effective. Recently, we have obtained crystals of the title compound, a mixture of NDA and *N*-Methyl Pyrrolidone (NMP). We call this phenomenon adductive crystallization and intend to apply this crystallization technique to the purification of NDA.

NDA crystallizes in the triclinic space group P1 (Kaduk & Golab, 1999), while NMP crystlalizes in the monoclinc space group P2_1_/c (Müller *et al.*, 1996). There have also been some reports on the adductive crystallization of dicarboxylic acids and amides, such as Terephthalic acid (TA) and *N*,*N*-dimethylacetamide (Guo *et al.*, 2009) and TA and *N*,*N*-dimethylformamide (Dale & Elsegood, 2004).

The title compound crystallized in the orthorhombic space group Fdd2, and the molecular structure is shown in Fig. 1. The asymmetric unit contains one half-molecule of NDA and one molecule of NMP. The pyrrolidone group has an envelope conformation with atom C9 at the flap. The dihedral angle between the mean planes of the naphthalene ring of the NDA molecule and the pyrrolidone ring of the NMP molecule is 22.39 (15)°.

In the crystal the NDA and NMP molecules are linked by strong O—H···O hydrogen bonds and C-H···O interactions (Fig. 2 and Table 1).

### Experimental

The title compound was obtained by putting 0.1 g of Naphthalene-2,6-dicarboxylic acid (NDA) into 1 ml of *N*-Methyl Pyrrolidone (NMP) at room temperature and then leaving the mixture in the freezer, which was maintained at 255 K, for 72 h. During the process, we observed the gradual disappearance of the NDA powder and the appearance of colourless needle-like crystals of the title compound.

### Refinement

In the final cycles of refinement, in the absence of significant anomalous scattering effects, Friedel pairs were merged and Δf " set to zero. The H-atoms were placed in calculated positions and were refined using a riding model: O—H = 0.82 Å, C—H_aromatic_ = 0.93 Å, C—H_alkyl_ = 0.97 Å, C—H_methyl_ = 0.96 Å, with *U*_iso_(H) = k × *U*_eq_(O or C), where k = 1.5 for CH_3_ H-atoms and k = 1.2 for all other H-atoms.

### Crystal data

**Table:**

C_12_H_8_O_4_·2C_5_H_9_NO	*F*(000) = 1760
*M**_r_* = 414.45	*D*_x_ = 1.349 Mg m^−^^3^
Orthorhombic, *F**d**d*2	Mo *K*α radiation, λ = 0.71073 Å
Hall symbol: F 2 -2d	Cell parameters from 1127 reflections
*a* = 19.7306 (11) Å	θ = 3.1–29.2°
*b* = 28.7632 (19) Å	µ = 0.10 mm^−^^1^
*c* = 7.1906 (4) Å	*T* = 120 K
*V* = 4080.8 (4) Å^3^	Needle, colourless
*Z* = 8	0.30 × 0.11 × 0.10 mm

### Data collection

**Table:**

Oxford Diffraction Xcalibur Atlas Gemini ultra diffractometer	1017 independent reflections
Radiation source: fine-focus sealed tube	847 reflections with *I* > 2σ(*I*)
graphite	*R*_int_ = 0.037
Detector resolution: 10.3592 pixels mm^-1^	θ_max_ = 25.4°, θ_min_ = 3.1°
ω scans	*h* = −23→19
Absorption correction: multi-scan (*CrysAlis PRO*; Oxford Diffraction, 2009)	*k* = −22→34
*T*_min_ = 0.987, *T*_max_ = 0.990	*l* = −8→7
3255 measured reflections	

### Refinement

**Table:**

Refinement on *F*^2^	Primary atom site location: structure-invariant direct methods
Least-squares matrix: full	Secondary atom site location: difference Fourier map
*R*[*F*^2^ > 2σ(*F*^2^)] = 0.037	Hydrogen site location: inferred from neighbouring sites
*wR*(*F*^2^) = 0.087	H-atom parameters constrained
*S* = 1.05	*w* = 1/[σ^2^(*F*_o_^2^) + (0.0405*P*)^2^ + 1.8717*P*] where *P* = (*F*_o_^2^ + 2*F*_c_^2^)/3
1017 reflections	(Δ/σ)_max_ < 0.001
138 parameters	Δρ_max_ = 0.17 e Å^−^^3^
1 restraint	Δρ_min_ = −0.20 e Å^−^^3^

### Special details

**Table:**

Experimental. Empirical absorption correction using spherical harmonics, implemented in SCALE3 ABSPACK scaling algorithm; CrysAlis PRO (Oxford Diffraction, 2009).
Geometry. Bond distances, angles etc. have been calculated using the rounded fractional coordinates. All su's are estimated from the variances of the (full) variance-covariance matrix. The cell esds are taken into account in the estimation of distances, angles and torsion angles
Refinement. Refinement of *F*^2^ against ALL reflections. The weighted *R*-factor *wR* and goodness of fit *S* are based on *F*^2^, conventional *R*-factors *R* are based on *F*, with *F* set to zero for negative *F*^2^. The threshold expression of *F*^2^ > σ(*F*^2^) is used only for calculating *R*-factors(gt) *etc*. and is not relevant to the choice of reflections for refinement. *R*-factors based on *F*^2^ are statistically about twice as large as those based on *F*, and *R*- factors based on ALL data will be even larger.

### Fractional atomic coordinates and isotropic or equivalent isotropic displacement parameters (Å^2^)

**Table:**

	*x*	*y*	*z*	*U*_iso_*/*U*_eq_	
O1	0.24703 (10)	0.09264 (7)	0.7871 (3)	0.0278 (7)	
O2	0.13547 (10)	0.10088 (8)	0.7582 (4)	0.0385 (8)	
C1	0.27857 (13)	0.23501 (10)	0.7441 (4)	0.0168 (8)	
C2	0.26776 (13)	0.18651 (10)	0.7497 (4)	0.0180 (8)	
C3	0.20359 (13)	0.16856 (10)	0.7495 (4)	0.0177 (8)	
C4	0.14695 (13)	0.19829 (10)	0.7411 (4)	0.0200 (9)	
C5	0.15563 (13)	0.24549 (10)	0.7410 (4)	0.0191 (9)	
C6	0.19144 (14)	0.11756 (10)	0.7638 (4)	0.0200 (8)	
O3	0.23717 (10)	0.00444 (7)	0.8202 (3)	0.0252 (7)	
N1	0.18459 (12)	−0.06402 (9)	0.8830 (4)	0.0223 (7)	
C7	0.18914 (15)	−0.01786 (11)	0.8877 (4)	0.0219 (9)	
C8	0.12795 (15)	0.00109 (11)	0.9868 (5)	0.0252 (9)	
C9	0.07849 (14)	−0.03970 (11)	0.9918 (5)	0.0259 (10)	
C10	0.12448 (14)	−0.08267 (11)	0.9734 (5)	0.0256 (10)	
C11	0.23599 (16)	−0.09402 (11)	0.8060 (5)	0.0294 (10)	
H1	0.23680	0.06520	0.80000	0.0420*	
H2	0.30480	0.16650	0.75370	0.0220*	
H4	0.10350	0.18580	0.73570	0.0240*	
H5	0.11800	0.26490	0.73890	0.0230*	
H8A	0.10880	0.02720	0.91940	0.0300*	
H8B	0.13950	0.01110	1.11160	0.0300*	
H9A	0.04650	−0.03800	0.88950	0.0310*	
H9B	0.05360	−0.04030	1.10810	0.0310*	
H10A	0.10330	−0.10650	0.89760	0.0310*	
H10B	0.13540	−0.09560	1.09430	0.0310*	
H11A	0.21610	−0.11380	0.71350	0.0440*	
H11B	0.27090	−0.07550	0.75000	0.0440*	
H11C	0.25520	−0.11270	0.90320	0.0440*	

### Atomic displacement parameters (Å^2^)

**Table:**

	*U*^11^	*U*^22^	*U*^33^	*U*^12^	*U*^13^	*U*^23^
O1	0.0248 (11)	0.0165 (11)	0.0421 (14)	−0.0012 (9)	0.0016 (10)	0.0040 (11)
O2	0.0264 (12)	0.0216 (12)	0.0675 (17)	−0.0061 (10)	−0.0131 (13)	0.0029 (13)
C1	0.0178 (13)	0.0197 (15)	0.0130 (13)	−0.0010 (12)	0.0015 (13)	0.0011 (14)
C2	0.0217 (14)	0.0186 (15)	0.0138 (14)	0.0029 (12)	0.0002 (13)	−0.0012 (14)
C3	0.0199 (15)	0.0193 (15)	0.0138 (14)	−0.0001 (13)	−0.0024 (12)	−0.0009 (14)
C4	0.0165 (14)	0.0266 (18)	0.0170 (14)	−0.0037 (13)	−0.0015 (14)	0.0019 (14)
C5	0.0178 (14)	0.0213 (17)	0.0183 (14)	0.0027 (12)	−0.0011 (13)	0.0007 (14)
C6	0.0214 (14)	0.0187 (15)	0.0200 (15)	−0.0002 (14)	−0.0025 (13)	0.0012 (14)
O3	0.0240 (12)	0.0195 (11)	0.0322 (13)	−0.0027 (10)	0.0047 (10)	0.0025 (11)
N1	0.0213 (12)	0.0209 (13)	0.0247 (13)	0.0002 (12)	0.0021 (11)	0.0037 (12)
C7	0.0235 (15)	0.0213 (16)	0.0209 (15)	0.0002 (15)	−0.0058 (13)	0.0006 (14)
C8	0.0256 (15)	0.0263 (17)	0.0238 (16)	0.0049 (14)	0.0001 (13)	−0.0018 (17)
C9	0.0229 (15)	0.0301 (19)	0.0246 (16)	0.0012 (14)	0.0011 (15)	0.0034 (16)
C10	0.0250 (16)	0.0281 (18)	0.0237 (17)	−0.0052 (15)	0.0004 (13)	0.0065 (15)
C11	0.0313 (18)	0.0259 (18)	0.0309 (17)	0.0064 (15)	0.0019 (15)	0.0017 (16)

### Geometric parameters (Å, °)

**Table:**

O1—C6	1.321 (3)	C4—H4	0.9300
O2—C6	1.205 (3)	C5—H5	0.9300
O1—H1	0.8200	C7—C8	1.504 (4)
O3—C7	1.243 (4)	C8—C9	1.527 (4)
N1—C7	1.331 (4)	C9—C10	1.539 (4)
N1—C10	1.455 (4)	C8—H8A	0.9700
N1—C11	1.442 (4)	C8—H8B	0.9700
C1—C5^i^	1.414 (4)	C9—H9A	0.9700
C1—C1^i^	1.419 (4)	C9—H9B	0.9700
C1—C2	1.412 (4)	C10—H10A	0.9700
C2—C3	1.367 (4)	C10—H10B	0.9700
C3—C6	1.490 (4)	C11—H11A	0.9600
C3—C4	1.409 (4)	C11—H11B	0.9600
C4—C5	1.368 (4)	C11—H11C	0.9600
C2—H2	0.9300		
			
C6—O1—H1	109.00	C7—C8—C9	104.2 (3)
C10—N1—C11	121.6 (3)	C8—C9—C10	103.8 (2)
C7—N1—C10	114.3 (2)	N1—C10—C9	102.9 (2)
C7—N1—C11	124.0 (3)	C7—C8—H8A	111.00
C2—C1—C5^i^	122.1 (2)	C7—C8—H8B	111.00
C1^i^—C1—C5^i^	119.2 (3)	C9—C8—H8A	111.00
C1^i^—C1—C2	118.7 (2)	C9—C8—H8B	111.00
C1—C2—C3	120.9 (2)	H8A—C8—H8B	109.00
C4—C3—C6	118.2 (2)	C8—C9—H9A	111.00
C2—C3—C6	121.4 (2)	C8—C9—H9B	111.00
C2—C3—C4	120.4 (3)	C10—C9—H9A	111.00
C3—C4—C5	120.2 (2)	C10—C9—H9B	111.00
C1^i^—C5—C4	120.6 (2)	H9A—C9—H9B	109.00
O1—C6—O2	123.3 (3)	N1—C10—H10A	111.00
O2—C6—C3	122.5 (3)	N1—C10—H10B	111.00
O1—C6—C3	114.2 (2)	C9—C10—H10A	111.00
C3—C2—H2	120.00	C9—C10—H10B	111.00
C1—C2—H2	120.00	H10A—C10—H10B	109.00
C3—C4—H4	120.00	N1—C11—H11A	109.00
C5—C4—H4	120.00	N1—C11—H11B	110.00
C4—C5—H5	120.00	N1—C11—H11C	109.00
C1^i^—C5—H5	120.00	H11A—C11—H11B	109.00
O3—C7—N1	123.8 (3)	H11A—C11—H11C	110.00
O3—C7—C8	127.6 (3)	H11B—C11—H11C	109.00
N1—C7—C8	108.6 (3)		
			
C10—N1—C7—O3	179.0 (3)	C1—C2—C3—C4	0.9 (4)
C10—N1—C7—C8	−0.7 (4)	C2—C3—C6—O1	3.7 (4)
C11—N1—C7—O3	2.7 (5)	C2—C3—C4—C5	−2.8 (4)
C11—N1—C7—C8	−177.1 (3)	C6—C3—C4—C5	175.4 (3)
C7—N1—C10—C9	15.8 (4)	C4—C3—C6—O2	4.1 (4)
C11—N1—C10—C9	−167.8 (3)	C2—C3—C6—O2	−177.7 (3)
C2—C1—C5^i^—C4^i^	−178.1 (3)	C4—C3—C6—O1	−174.5 (3)
C2—C1—C1^i^—C5	−2.9 (4)	C3—C4—C5—C1^i^	1.8 (4)
C1^i^—C1—C2—C3	1.9 (4)	O3—C7—C8—C9	165.4 (3)
C5^i^—C1—C2—C3	−178.9 (3)	N1—C7—C8—C9	−14.9 (3)
C2—C1—C1^i^—C2^i^	176.3 (3)	C7—C8—C9—C10	23.5 (3)
C5^i^—C1—C1^i^—C5	177.9 (3)	C8—C9—C10—N1	−23.5 (3)
C1—C2—C3—C6	−177.3 (3)		

Symmetry codes: (i) −*x*+1/2, −*y*+1/2, *z*.

### Hydrogen-bond geometry (Å, °)

**Table:**

*D*—H···*A*	*D*—H	H···*A*	*D*···*A*	*D*—H···*A*
O1—H1···O3	0.82	1.75	2.556 (3)	165
C2—H2···O2^ii^	0.93	2.48	3.163 (4)	131
C8—H8A···O2	0.97	2.47	3.311 (4)	145

Symmetry codes: (ii) *x*+1/4, −*y*+1/4, *z*+1/4.

## References

[bb1] Dale, S. H. & Elsegood, M. R. J. (2004). *Acta Cryst.* C**60**, o444–o448. 10.1107/S010827010400976X 15178875

[bb2] Dolomanov, O. V., Bourhis, L. J., Gildea, R. J., Howard, J. A. K. & Puschmann, H. (2009). *J. Appl. Cryst.* **42**, 339–341.

[bb3] Guo, X., Cheng, Y. & Li, X. (2009). *Acta Cryst.* E**65**, o1794. 10.1107/S1600536809025793 PMC2977459 21583500

[bb4] Kaduk, J. A. & Golab, J. T. (1999). *Acta Cryst.* B**55**, 85–94. 10.1107/s0108768198008945 10927342

[bb5] Müller, G., Lutz, M. & Harder, S. (1996). *Acta Cryst.* B**52**, 1014–1022.

[bb6] Nagase, Y., Yamamoto, K., Tanaka, T. & Hamaguchi, M. (2004). US Patent No. 6756509.

[bb7] Oxford Diffraction (2009). *CrysAlis PRO* Oxford Diffraction Ltd, Yarnton, England.

[bb8] Sheldrick, G. M. (2008). *Acta Cryst.* A**64**, 112–122. 10.1107/S0108767307043930 18156677

